# Factors associated with exclusive breastfeeding practices in Debre Berhan District, Central Ethiopia: a cross sectional community based study

**DOI:** 10.1186/s13006-015-0049-2

**Published:** 2015-08-13

**Authors:** Maeza Mitiku Asfaw, Mesele Damte Argaw, Zelalem Kebede Kefene

**Affiliations:** Addis Ababa City Government Health Bureau, Addis Ketema Sub City Health Department, Addis Ababa, Ethiopia; Private Health Sector Program, P O Box 2372, Code 1250 Addis Ababa, Ethiopia; Malaria Consortium, Addis Ababa, Ethiopia

## Abstract

**Background:**

Globally, an estimated 6.3 million children under-five years of age died in the year 2013. To reduce the burden of morbidity and mortality of infants, Ethiopia adopted the World Health Organization (WHO) recommendation of exclusive breastfeeding (EBF) for the first six months of life. The objective of this study was to assess factors associated with EBF practices among mothers who have an infant aged below 12 months in *Debre Berhan* District, Ethiopia.

**Methods:**

In this study we employed a cross sectional community based quantitative survey method, and data were collected from January through April 2014. Univariate statistical analysis was used to describe variables using frequencies and percentages. Multivariable logistic regression model was developed; the factors associated with EBF practice were identified.

**Result:**

We enrolled 634 mothers with their index infant aged under 12 months. Four hundred thirty five (68.6 %; 95 % CI: 64.9 %, 72.2 %) mothers practiced EBF to six months. In this study 83.4 % of mothers were knowledgeable with the recommended duration of EBF. About 97.5 % of mothers had a positive attitude towards EBF practice. Mothers from rural places were 4.54 times likely to EBF than mothers residing in urban areas Adjusted Odds Ratio (AOR 4.54; 95 % CI: 2.64, 7.81, *p* = 0.001). The odds of mothers aged 25 to 35 years to practice EBF was up to 8.9 times more than mothers aged less than 25 years (AOR 8.99; 95 % CI: 4.56, 17.73, *p* = 0.001). The odds of mothers who didn’t receive infant feeding counselling was 0.42 times less likely to practice EBF than those who received Counselling services (AOR 0.42; 95 % CI: 0.24, 0.73, *p* = 0.002). Employed mothers were found to be 0.36 times less likely to practice EBF than housewives (AOR 0.36; 95 % CI: 0.18, 0.73, *p* = 0.005). Household income did not show a statistically significant association with EBF.

**Conclusions:**

The knowledge and attitude of mothers towards EBF was found to be very high. In this study, two thirds of mothers practiced exclusive breastfeeding. Improving access to information on recommended infant feeding during routine maternal and child health services is recommended. It is important to encourage EBF among the young, employed and urban mothers through proper counselling and mother friendly work environment.

## Background

Globally, there has been progress in improving child survival. Though the world promised to reduce child death by two thirds by 2015 from the level recorded in 1990, in the last two decades the under-five deaths was reduced by half in 2013, from 12.7 to 6.3 million. To keep the momentum of these achievements nearly 180 governments have pledged to scale up efforts to accelerate the reduction in maternal, neonatal and child deaths as part of achieving the Millennium Development Goal (MDG) [1, 2].

Sub-Saharan Africa (SSA) continues to shoulder the greatest burden where the under-five deaths are 15 times higher than deaths in an average high income country [1]. Ethiopia is one of the countries with the highest infant mortality rate (59/1000 live birth) [3]. The country has developed a strategic document to systematically address issues regarding child survival, infant and young children feeding [4, 5]. To reduce the burden of morbidity and mortality of infants, Ethiopia adopted the World Health Organization (WHO) recommendations to provide exclusive breastfeeding (EBF) for infants in the first 6 months of life [6].

The trends of EBF from 1995 to 2010 were estimated using global database of 140 countries from United Nations Children’s Fund and they found that in spite of the facts known about EBF, it was not widely practiced in many developing countries [7]. The situation in Ethiopia is not different from many countries. Evidence revealed that though 96 % of children had ever breastfed in their lifetime [8] a significant proportion of them had delayed initiation of breastfeeding [9] or were given a prelacteal meal, and were subjected to early weaning with associated health risks for the infants.

Several studies documented that the prevalence of EBF in Amhara Region of Ethiopia remains close to 50 per cent [10–13]. Socio-demographic characteristics including maternal age, educational status, employment and household income were identified as factors influencing EBF practice [10–15]. The routine maternal and child health services (antenatal, delivery, postnatal) and infant feeding counselling were positively associated with EBF. Psycho-social support offered for mothers, general feeding habit of the community and gender were found to have significant associations with better EBF practices [10, 16–19]. Mothers with a desirable knowledge level, positive attitude and experiences of EBF were identified as immediate determinants for maintaining EBF practice [11, 13, 17, 20]. These factors have been identified as a predictor for EBF practice with various levels of incidence and strengths. Hence, it is difficult to generalize the findings for other similar populations.

In 2003 the Federal Ministry of Health of Ethiopia (FMOH) and development partners designed Health Extension Program (HEP). The main purpose of the HEP includes production of health at household level and improving access to basic health services at the community and household level. Its goal is to promote appropriate health behaviour and outcomes of maternal, neonatal and child health services [21, 22]. The community health program is led and executed by Health Extension Workers (HEWs) and the support they received from community health agents like Health Development Army (HAD) [23]. HEWs and the HDAs are expected to improve the level of awareness about newborn, infant and child feeding issues in the community. One of the goals illustrated in the strategic document is to improve the rate of exclusive breastfeeding to increase from 49 % in 2010 to 70 % by the year 2015 [20].

*Debre Berhan* District has executed the complete set of HEP. The main focus of the district was to further reduce the infant mortality rate. The district wants to assess the infant EBF practice of the mother and factors influencing for the desired practice [24]. This community based cross sectional study was conducted in *Debre Berhan* District, in North Shoa Administrative Zone of Amhara Region. The aim of the study was to determine the knowledge, attitude and practice towards EBF among mothers who have children aged below 12 months and to explore factors influencing EBF practice. The purpose of the study was to assess the incidence of EBF in *Debre Berhan* District and recommend appropriate interventions to improve its outcome. The following hypotheses were put forth: there was no difference on EBF practice based on socio-demographic characteristics of mothers. The incidence of EBF for the first six months was low among mothers having children under twelve months of age.

### Definition of terms

According to WHO [6], exclusive breastfeeding is the practice of feeding breast milk only, including expressed breast milk, to infants and excluding water, other liquids, breast milk substitutes, and solid foods. Vitamin drops, minerals, oral rehydrating solution (ORS) and medicines may be given. Prelacteal feedings are defined as feedings that are given to infant before they are put on the breast for the first time. HEWs: A community level health worker trained for one year at an undergraduate level to deliver preventive, promotive and curative health services, such as maternal and child health services [21]. Health Development Army: In a network of up to 5 families each, one of the families, who is an innovator or front liner in practicing health behaviour, leads the network and gradually influences the rest of the households to acquire skill and changes in attitudes towards healthy behaviour. The network is technically supported by the HEWs, who facilitate and follow up regularly conversations within the community. Kebele: The smallest administrative unit with 5000 people [23].

## Methods

### Study setting

The study was conducted in *Debre Berhan* District, one of the 177 districts of Amhara Region of Ethiopia. The district is located 125 km to the North of Addis Ababa, the capital city of Ethiopia. *Debre Berhan* district has nine *kebele*s, of which five *kebeles* are categorized as urban and the remaining four are rural. Based on the projection of the 2007 population census in the year 2014, the district has an estimated 84,920 (41,187 males and 43,733 females) inhabitants, of which 2,649 are infants. There are ten health extension posts and five health centres with at potential health service coverage of 100 % [24].

### Study design and sample size

A cross sectional community based survey was conducted among mothers with their index infant age less than 12 months from January through April 2014, in *Debre Berhan* District, of North *Shoa* Administrative Zone, in Amhara Region. The sample size was determined using Cochran’s formula [25]:

$$ ni=\frac{{Z^2}_{\raisebox{1ex}{$\alpha $}\!\left/ \!\raisebox{-1ex}{$2$}\right.}p\left(1-p\right)}{d^2} $$

Where, ni is the sample size, Z is standard normal variable at 95 % Confidence Interval (1.96), P is (0.52) the proportion of mothers who practiced exclusively breastfeeding for their infants in Ethiopia [8], d is the marginal error (0.05), design effect (1.5) and contingency for non-response (0.10).

### Sampling

First we selected four (2 urban and 2 rural) out of nine *kebeles*, the smallest administrative units, using lottery methods. Then, we conducted population census in these *kebeles* and identified 1177 mother with index infant aged less than 12 months. Sampling frame was developed and study subjects were selected every other household with systematic random sampling techniques. During data collection 53 mothers and their index infants were replaced by the next study subject due to incomplete responses and missing basic information. Data were collected from 634 mothers with their index infant aged less than 12 months. At the end of the data collection the recommended infant feeding practices were delivered to all mothers as key health messages.

In this study we included mothers who lived in the study area for more than six months, with one infant at the time of the survey and who have no restrictions to breastfeed their index infant due to serious medical or surgical health conditions. Furthermore, mothers who were pregnant at the time of the survey, were excluded. In the case of having two or more mothers with infants in the household we selected the one with younger age infant.

### Measures

The data were collected using interview method by pretested questionnaires. The questionnaire was prepared first in English and then translated into Amharic. To maintain the consistency of the tool it was back translated to English by two different public health professionals. The data collectors and their supervisors attended the necessary training on the objective of the study, data collection techniques, procedures and instruction to complete the questionnaires. The tool was pretested with 70 mothers who had infants under one year old in *Basona Worana* District of North Shoa Administrative Zone, which was not the part of our study area. Then the data collection tools were amended and rearranged to accommodate the desired data for the study.

The data were collected using a tool designed and piloted by the researchers [8]. The data were collected by volunteers who have completed 10^th^ grade education. The supervisors were two health professionals with a Masters degree in Public Health. The data collection team were trained and the data collection process was supervised by the researchers.

The questionnaires have socio-demographic information of mothers and infants, knowledge, attitude and practice of mothers on exclusive breastfeeding. In this study the dependent variable was EBF Practice [6] for the first six month of the index infant by mothers. In the regression analysis EBF practice was coded as ‘1’ while ‘0’ was coded for non-EBF practices.

The independent variable were age, educational status, residence, marital status of mother, household income, occupation, family size, birth order, sex of infant, place of delivery, antenatal and postnatal service uptake. To estimate the knowledge of mothers on EBF, we asked mothers about ideal duration of EBF and the time to wean off infants. In this study, those mothers who correctly identified the duration of EBF as six months were considered as knowledgeable. Five questions were presented to mothers to identify their attitudes towards EBF practice. Mothers who want to EBF their next baby or those who encourage their peers to do so were considered to have a favourable attitude towards EBF. The practice of mothers were measured based on their report on initiation time of breastfeeding, provision of colostrum, and the duration of EBF for the index infant. Mothers who fed their infant only breast milk for the first six months of life were considered as having an ideal practice.

The age of mothers were categorized into three groups i.e. < 25, 25–35 and > 35 years. The younger age group was taken as a reference population in the regression analysis. Religion of mothers was coded as ‘0’ for Christian and ‘1’ for Muslim. Urban and rural residences of mothers were coded as ‘0’ and ‘1’ respectively. With regard to educational status of parents those who could not read and write were coded ‘0’ while the rest were coded as ‘1’. Mothers who were reported as housewife were coded ‘0’ while farmers and employed mothers were coded ‘1’. The lowest household income was coded ‘0’ while the other two levels were coded ‘1’. Mothers who received infant feeding counselling and delivered in health institution were coded as ‘0’, their counter parts coded as ‘1’.

### Statistical analysis

The data were checked for completeness, consistency and cleaned manually. It was entered into EPI Info version 3.5.3.(CDC, Atlanta, Georgia) statistical software and exported to Statistical Package for Social Science (SPSS) research IBM version 20.0 for Window, (IBM SPSS Version 20.0. Armonk, NY: IBM Corp). The descriptive finding was presented with tables and graphs. Univariable statistical tests were computed to identify all possible predictor variables. Then the multi-variable logistic regression model was developed considering EBF practice as dependent and those nominee variables selected based on the result of the uni-variable test statistics which had a Wald Statistics with *p-*value < 0.20. This decision was made based on suggestion to capture all important variables made by Bendel and Afiff [26]. Results are reported as Crude Odds Ratio (COR) or Adjusted Odds Ratio (AOR) with 95 % Confidence Intervals (CIs). The statistical significance tests was accepted at *p* < 0.05.

### Ethical approval

Ethical clearance was obtained from Institute Review Board (IRB) of *Debre Berhan* University. Permission was granted from *Debre Berhan* District Health Office and Kebele Administrations. Verbal consent was taken from all mothers who participating in the study.

## Results

A completed data were obtained from 634 mothers and their index infant aged below 12 months. One half, 317 (50.0 %) of the study subjects were residing in rural *kebeles*. The majority (90.5 % ) were Christians. One fourth of mothers (25.7 %) were illiterate, and 9.5 % of husbands were unable to read and write. Close to one third (32.8 %) of mothers were farmers. The majority of mothers 353 (55.7 %) were in the age category between 25 and 35 years. The mean age with (±SD) of respondent mothers was 30.9 (±6.2) years, which ranges from 17 to 48 years. The mean (±SD) age of the index infants was 7.7 ± 3.2 months. In 2014 the lowest daily wage for unskilled government worker in Ethiopia was 19.00 ETB (0.95 USD) which was equivalent to 420.00 ETB (21.00 USD) per month [27]. One third of (34.2 %) households had an income of less than 30.00 USD per month (Table 1).

**Table 1:** Socio-demographic characteristics of study participants mothers (*n* = 634) Debre Berhan District, Ethiopia, April 2014

Characteristics	Frequency	Percentage
Place of residence		
Urban	317	50.0
Rural	317	50.0
Age of mothers (years)		
<25	108	17.0
25–35	353	55.7
>35	173	27.3
Religion		
Christian	574	90.5
Muslim	60	9.5
Educational status of mother respondents		
Unable to read and write	163	25.7
Read and write	180	28.4
Elementary school	105	16.6
High school & preparatory school	103	16.2
Graduate	83	13.1
Educational status of husband		
Unable to read and write	60	9.5
Read and write	173	27.3
Elementary school	106	16.7
High school & preparatory school	122	19.2
Graduate	173	27.3
Current working status of respondents		
Farmer	208	32.8
Civil servant	130	20.5
Housewife	125	19.7
Merchant	107	16.9
Daily Labourer	46	7.3
Student	11	1.7
House servant (maid)	7	1.1
Income of the household		
Less than 30.00 USD^¥^	217	34.2
30.00–60.00 USD	175	27.6
Greater than 60.00 USD	242	38.2
Family size (mean ± Sd) (Persons/household)	4.48 ± 1.57	
Sex of infant		
Male	344	54.3
Female	290	45.7
Age of infant (mean ± Sd ) in months	7.79 ± 3.23	

NB: ^¥^ 1.00 USD = 19.00 ET Birr

### Maternal, neonatal and child health care services

Five hundred sixty seven (89.4 %) of mothers had received antenatal care (ANC) services during their last pregnancy. Among mothers 16 (2.8 %) had single visit. 230 (40.6 %) had two or three and the rest 321 (56.6 %) had four or more visits. More than one third (36.9 %) were primipara mothers, below half of them (44.2 %) were multipara mothers and the rest (18.9 %) were grand multipara mothers.

The majority of mothers (84.4 %) delivered their index infant in the health institutions and were attended by trained health workers. More than one tenth (12.0 %) of the deliveries were attended by Traditional Birth Attendants (TBAs) and the rest 3.6 % of mothers received delivery assistance from HEWs. Sixty two point six (62.6) per cent of mothers sought postnatal care after their most recent deliveries. Infant feeding counselling services were received by 82.6 % of mothers during the antenatal and postnatal care services.

### Knowledge and attitude of mothers towards EBF

The majority (97.5 %) of mothers reported that they had ever heard about EBF from one or more sources. The main sources of information for mothers on EBF (90.5 %) were reported as HEWs or Health Workers, followed by mass media (radio or television) (30.4 %). Eighty three per cent of mothers correctly identified the ideal duration of EBF as from birth to six month of life of an infant. The reasons cited by mothers for practicing breastfeeding for infants include: 71.1 % believed it is a societal norm, 29.2 % thought of it as natural gift, and 20.5 % consider breastfeeding as a clean and accessible meal for infants. The majority (89.9 %) of mothers said they would breastfeed their next infant. Almost all (97.5 %) mothers encourage their peers to practice EBF. Their main reason for encouraging others were: 68.1 % believe that breastfeeding prevent infection and infant death, 36.9 % recommend it for improving infant wellbeing and 19.6 % recommend it as a cost effective method of infant feeding practice (Table 2).

**Table 2:** Knowledge and attitude of mothers (*n* = 634) towards exclusive breastfeeding (EBF), Debre Berhan District, April 2014

Characteristics	Frequency	Percentage
Knowledge		
Ever heard about exclusive breastfeeding		
Yes	618	97.5
No	16	2.5
If you have heard about exclusive breastfeeding, from whom (*n* = 618)^¥^		
Health Extension Workers / Health Workers	559	90.5
Mass media (radio or television)	188	30.4
Husband	151	24.4
Friends/colleagues	114	18.4
Health Development Army	60	9.7
Community Health Agents	59	9.5
Ideal time duration for EBF		
correct time (6 months )	529	83.4
Incorrect time (less or greater than 6 months)	105	16.6
Ideal time to wean an infant		
correct time (6 months )	453	71.5
Incorrect time	181	28.5
Attitude		
Why do you breastfeed your infant (*n* = 634) ^¥^		
An accepted norm to EBF infant	451	71.1
It is natural gift	185	29.2
it is clean and accessible	130	20.5
Easy and cost effective	68	10.7
Do you want to breastfeed your next baby?		
Yes	519	81.9
No	25	3.9
Don’t want any more children	90	14.2
Do you encourage mothers to EBF their infant?		
yes	618	97.5
No	16	2.5
Why do you encourage exclusive breastfeeding? (*n* = 634) ^¥^		
Prevent infection and infant death	421	68.1
Improve infant's strength	228	36.9
Cost effective	121	19.6

^¥^- sum greater than hundred due to multiple responses

### Practice of mothers towards EBF

Two thirds, 448 (70.7 %) of mothers had initiated breastfeeding within one hour while it improved to 488 (77.0 %) during the first six hours after delivery. Five hundred and five (79.9 %) mothers reported that they fed the colostrum to their index infants. However, 90 (14.2 %) of mothers also reported giving a prelacteal meal for their infants. Of these 53.3 % gave butter, 40.0 % gave a mixture of water with sugar and 6.3 % fed cow’s milk to their infants.

Two thirds of mothers 435 (68.6 % with 95 % CI: 64.9–72.2 %) had reported practicing EBF for their index infants. Out of 169 mothers who initiated early weaning, the food they provided for their infant before 6 months includes cow’s milk (45.0 %), and formula milk (21.3 %). The reason for cessation of EBF for mothers were because they needed to return back to work after completing two months of maternity leave (30.5 %), they believed their breast had insufficient breast milk (23.9 %), they introduced supplementary food to correct poor infant growth for age (13.2 %), had maternal health problem (12.2 %), they thought they had fed for recommended period (9.1 %) and the rest 8.1 % reported desire of infant for additional meal.

### Factors associated with exclusive breastfeeding practice

In this study we assessed the factors associated with EBF practice of mothers. A univariate logistic regression statistical test was done and the presence of any relationship between independent factors with EBF practice of mothers as a dependent variable was examined. The odds of EBF (*p* < 0.05) for rural resident mothers was 3 fold higher (COR 3.13; 95 % CI: 2.19, 4.47, *p* = 0.001), 10 fold for mothers aged between 25 and 35 years (COR 10.52; 95 % CI: 5.71, 19.35, *p* = 0.001), than their counterparts. Women who completed secondary and tertiary education had lower odds of reporting EBF practices when compared with their counterparts who were illiterate, COR (95 % CI): 0.38 (0.20, 0.68) and 0.44 (0.24, 0.79), respectively. The Odds of EBF for housewife 1.6 times higher than employed mothers (COR 1.66; 95 % CI: 1.136, 2.41, *p* = 0.01), however there was no statistically significant difference with farmers (COR 1.06; 95 % CI: 0.64, 1.75, *p* = 0.80). The odds of EBF practice was 0.60 times lower among mothers with household income 30 to 60 (USD) than their counterpart mothers with household income less than 30.0 (COR 0.60; 95 % CI: 0.40, 0.89, *p* = 0.013). Mothers who did not receive infant feeding counselling (COR 0.47; 95 % CI: 0.31, 0.72, *p* = 0.001) and did not deliver in the health institution (COR 0.53; 95 % CI: 0.34, 0.83, *p* = 0.005) had lower odds of reporting EBF than those who exhibited these characteristics. Factors like father’s educational status, family size, age of infants, sex of infants, birth orders, birth intervals, and parity of mothers didn’t reveal statistically significant association (*p* > 0.05) with the EBF practice of mothers.

After identifying the nominee predictor variables of the EBF practice of mothers using univariable logistic regression statistical test, we developed a model to control the possible confounder variables and identified the real predictor variables.

The odds of EBF practice mothers from rural area was 4.54 times higher than urban resident mothers (AOR 4.54; 95 % CI: 2.64, 7.81, *p* = 0.001). The odds of EBF practice among mothers age 25–35 years and greater or equal to 36 years was up to nine fold (AOR 8.99; 95 % CI: 4.56, 17.73, *p* = 0.001) and 3.6 fold (AOR 3.60; 95 % CI: 2.03, 6.38, *p* = 0.001) higher than their counterpart mother aged less than 25 years. Mothers who couldn’t read and write and who were housewives in occupation had a higher odds of EBF practice than their counterparts respectively (*p* = 0.001). But household income didn’t show statistically significant association with EBF practices of mothers (*p* > 0.05) (Table 3).

**Table 3:** Univariate and multivariable logistics regression analysis of socio-demographic and economic variables exclusive breastfeeding in Debre Berhan District, Ethiopia, April 2014

Characteristics	Exclusive breastfeeding		COR (95 % CI)	AOR (95 % CI)	*p*-value
	Yes	No			
	N (%)	N (%)			
Place of residence					
Urban	180 (41.4)	137 (67.2)	1	1	
Rural	225 (58.6)	62 (32.2)	3.13 (2.19, 4.47)	4.54 (2.64, 7.81)	0.001
Religion					
Christian	401 (92.2)	173 (86.9)	1	1	
Muslim	34 (7.8)	26 (13.1)	0.56 (0.32, 0.96)	0.75 (0.38, 1.46)	0.398
Age of mothers (years)					
<25	47 (10.8)	61 (30.7)	1	1	
25–35	234 (53.8)	119 (53.8)	10.52 (5.71, 19.35)	8.99 (4.56, 17.73)	0.001
>35	154 (35.4)	19 (9.5)	4.12 (2.43, 6.96)	3.60 (2.03, 6.38)	0.001
Educational status of mother					
Unable to read and write	132 (30.3)	31 (15.6)	1	1	
Read and write	138 (31.7)	42 (21.1)	0.16 (0.09, 0.29)	0.06 (0.02, 0.16)	0.001
Elementary school completed	68 (15.6)	37 (18.6)	0.21 (0.12, 0.37)	0.11 (0.04, 0.28)	0.001
High school completed	63 (14.5)	40 (20.1)	0.38 (0.20, 0.68)	0.22 (0.09, 0.53)	0.001
Graduate	34 (7.8)	49 (24.6)	0.44 (0.24, 0.79)	0.32 (0.16, 0.66)	0.002
Educational status of husband					
Unable to read and write	36 (8.3)	24 (12.1)	1	1	
Read and write	132 (30.3)	41 (20.6)	0.98 (0.54, 1.78)	3.03 (1.11, 8.24)	0.029
Elementary school completed	76 (17.5)	30 (15.1)	0.46 (0.28, 0.72)	2.09 (0.88, 5.00)	0.094
High school completed	88 (20.2)	34 (17.1)	0.58 (0.34, 0.97)	1.61 (0.70, 3.69)	0.254
Graduate	103 (23.7)	70 (35.2)	0.57 (0.34, 0.93)	1.26 (0.64, 2.48)	0.648
Current work status of mothers					
Housewife	91 (20.9)	34 (17.1)	1	1	
Working/employed	190 (43.7)	111 (55.8)	1.66 (1.13, 2.41)	0.36 (0.18, 0.73)	0.005
Farmer	154 (35.4)	54 (27.1)	1.06 (0.64, 1.75)	0.30 (0.14, 0.64)	0.002
Household income					
Less than 30USD	160 (36.8)	57 (28.6)	1	1	
30–60 USD	123 (28.3)	52 (26.1)	0.60 (0.40,0.89)	0.61 (0.32, 1.15)	0.129
Greater than 60 USD	152 (34.9)	90 (45.2)	071 (0.47, 1.08)	1.05 (0.58, 1.89)	0.858
Counselled on infant feeding					
Yes	375 (86.2)	149 (74.9)	1	1	
No	60 (13.8)	50 (25.1)	0.47 (0.31, 0.72)	0.42 (0.24, 0.73)	0.002
Place of delivery					
Health institution	379 (87.1)	156 (78.4)	1	1	
Home	56 (12.9)	43 (21.6)	0.53 (0.34, 0.83)	044 (0.25, 0.77)	0.004

NB: In multivariable logistic regression model variables entered: maternal age, religion, place of residence, educational status of mothers, educational status of fathers, occupational status of mothers, household income, infant feeding counselling and place of delivery

## Discussion

In this study, it was found that about 97.5 % of mothers had heard about EBF. The majority of mothers 83.4 % correctly knew that the ideal duration of EBF should be from the moment of a child's birth up to the first six months of life. This was higher than the findings of the studies in Kigali, Rwanda, and Kware town of Sokoto State, Nigeria. The first study found 74.4 % of refugee mothers in the urban area [28] and the second study, 31.0 % of women of child bearing age in small town were knowledgeable about the recommended EBF duration [29]. Moreover, a local study conducted in south west Ethiopia had revealed that about two thirds (67.0 %) of mothers were not knowledgeable on EBF [14]. This could have occurred due to the difference in the infant feeding culture and values of the community.

In this study we found that most (97.5 %) of the mothers had a positive attitude towards practicing EBF. This was in line with 90.1 % reported from mothers in Saudi Arabia who had a similar attitude towards EBF [17]. This could be attributable to counselling service mothers received on infant feeding at health facilities or at household level [23].

Two thirds (70.7 %) of mothers have initiated breastfeeding for their infants within one hour after delivery. This report was in line with 72.7 % similar practice in West Nepal [30]. The finding was found higher than the 31 % documented in Saudi Arabia [17]. Ethiopian Demographic and Health Survey (EDHS, 2011) reported 57 % in urban area versus 51 % in rural area initiating breastfeeding within an hour with the lowest among regions being Amhara Region with 37.5 % [8] and 53 % of mothers initiated breastfeeding within an half an hour time for their newborns in Nigeria [29]. However, this was much lower than the Ethiopian national goal of initiating breastfeeding within one hour for about 92.0 % of newborns, to be achieved by the year 2015 [20].

Mothers were requested by the data collector on their practice on dietary recall for the first six month of life of their index baby and two third (68.6 %) of mothers reported that they exclusively breastfed their infants during the first six months of life. This result was higher than 52.0 % for the nationwide prevalence of EBF documented in EDHS [8], 50.3 % in Bahir Dar Town [10], 51.9 % in Harer town [12] and 34.4 % in Kigali [28]. Moreover, it was much higher 8.2 to 12.2 % in Saudi Arabia [17, 31], but the finding was lower than the 71.3 % in Goba, South East Ethiopia [15]. In this study we enrolled all infant age less than 12 months, hence the point estimate might be inflated. Socio-cultural difference on newborns feeding, the intervention made to promote the EBF, and the improved HW assisted delivery might be the main reason for the observed difference in this study. The main reason for cessation of EBF before the age of six months was mothers who need to return back to work (30.5 %), this finding was similar with the one reported in Southwest Nigeria (24.0 %) [12, 31, 32].

Rural resident have 4.54 time higher chance of practicing EBF for their infant than their counter part urban resident mothers (*P* < 0.05). This finding was in line with the result of the studies where rural residents practiced EBF 1.6 to 2.2 times higher in rural than urban area in Malaysia and Saudi Arabia [31, 32]. This could be explained by the Ethiopian national labour law that grants mothers two months maternity leave. The short maternity leave and the unfavourable work condition to continue EBF might be the cause for early weaning practices of mothers.

Mothers whose age group ranges from 25 to 35 years were nine fold more likely to practice EBF than their counter part mothers who were younger than 25 years (*p* < 0.05). This finding was in agreement with the study in Hareri Regional State, of Ethiopia [12]. This could be due to the fact that younger age mothers do have a better job opportunity and lack the time to EBF their infant. In this study we found that when the educational status of mother was to the higher level, their chance of practicing EBF significantly reduced (*p* < 0.05). This could be due to the fact that educated mothers have better job opportunities than illiterate mothers, so they don’t have enough time to maintain EBF practice.

Mothers who are housewives were better at practicing EBF than employed mothers or farmers (*p* < 0.05). This result is consistent with study conducted in other parts of Ethiopia namely Bahir Dar and Goba District [10, 15]. A similar finding was documented in Saudi Arabia, Malaysia and Cambodia [31–33]. This could be universal due to the fact that mothers who have more time to be with their infants, have more opportunity to practice EBF than those who do not have due to work or other reasons.

Mothers who have received infant feeding counselling had a much higher adherence to practice EBF for the recommended duration than those mothers who didn’t receive the services (*p* < 0.05). There was a similar finding in a study conducted at Bahir Dar [10]. Institutional delivery had a positive association with EBF practice than their counter parts who delivered at home (*p* < 0.05). Similar report was documented in Bahir Dar and Harer town [10, 12]. And the main reason could be the access to infant feeding counselling service rather than number of visits.

Unlike other studies, in this study fathers educational status, age of infant, sex of infant, birth interval, birth order, parity of mothers, frequency of antenatal care services, frequency of postnatal care, partner’s encouragement and maturity was not found to have a statistically significant relationship with practicing EBF. In other studies, a female infant [16], younger age infant [10, 17] and married mothers [18, 19] had a higher chance of practicing EBF. The difference could be explained by the existing socio-cultural difference on infant feeding, sex preferences and partners involvement among study participants.

### Limitations of the study

*Debre Berhan* district is selected using purposive sampling methods so the findings cannot be generalized to other places. Since the study participants are mothers with a child aged less than 12 months, the estimation of EBF practice might be inflated by infant’s aged less than 6 months. There is also a possibility of recall bias on provision of prelacteal feeds and identifying the exact time of initiating weaning meals.

## Conclusions

In the studied area the knowledge and attitude of mothers towards EBF was found to be very high. Two third of mothers exclusively breastfed their infants. The main source of health information on EBF was health extension workers. Some of the factors contributing to higher EBF practice identified include being from rural *kebele*, maternal age greater or equal to 25 years, and housewives significantly adhered to the recommended duration. Moreover, provision of infant feeding counselling during maternal and child health services and delivery in institutions were factors found to be associated with improved EBF practice.

Maintaining access to information on infant feedings using the opportunity during maternal, neonatal and child health services, mass media and community health care system were recommended. Encouraging young, employed and urban resident mothers to practice EBF through counselling and preparing enabling environment is recommended.
